# Hospital-Acquired Disability as a Predictor of Functional Decline in ICU Survivors: A Multicenter Prospective Cohort Study in Japan

**DOI:** 10.3390/jcm14228168

**Published:** 2025-11-18

**Authors:** Yuki Iida, Shinichi Watanabe, Yorihide Yanagita, Ayato Shinohara, Tomoyuki Morisawa, Kengo Obata, Ryo Kozu, Shigeaki Inoue, Osamu Nishida, from the RELIFE Network

**Affiliations:** 1 Faculty of Health and Medical Sciences, Aichi Shukutoku University, Nagakute 480-1197, Japan; 2 Department of Physical Therapy, Faculty of Rehabilitation, Gifu University of Health Science, Gifu 500-8281, Japan; billabonghonor@yahoo.co.jp; 3 Department of Physical Therapy Science, Nagasaki University Graduate School of Biomedical Science, Nagasaki 852-8102, Japan; y-yanagita@nagasaki-u.ac.jp (Y.Y.);; 4 Department of Rehabilitation, Fujita Health University Hospital, Toyoake 470-1192, Japan; ayato@fujita-hu.ac.jp; 5 Department of Rehabilitation, Kobe Rehabilitation Hospital, Kobe 651-1106, Japan; t.morisawa@kzc.jp; 6 Department of Rehabilitation, Japanese Red Cross Okayama Hospital, Okayama 700-0941, Japan; 7 Department of Emergency and Critical Care Medicine, Wakayama Medical University, Wakayama 649-7113, Japan; 8 Department of Anesthesiology and Critical Care Medicine, Fujita Health University School of Medicine, Toyoake 470-1192, Japan

**Keywords:** post-intensive care syndrome, hospital-acquired disability, ICU rehabilitation, frailty, functional decline, Kihon Checklist, long-term outcomes

## Abstract

**Background:** Hospital-acquired disability (HAD), defined as a decline in activities of daily living (ADL) during hospitalization, is a significant component of post-intensive care syndrome (PICS) and may influence long-term outcomes in critically ill patients. Its impact on post-discharge functional recovery, especially among patients who appear ADL-independent at discharge, remains unclear. **Methods:** This analysis of the multicenter prospective J-RELIFE cohort included 357 ICU patients aged ≥ 40 years who required mechanical ventilation for ≥48 h. The primary outcome was global functional decline, defined as a Kihon Checklist (KCL) score ≥ 8 at 3 months after hospital discharge. Multivariable logistic regression and Cox proportional hazards models were used to identify independent predictors of functional decline, including HAD (Δ Barthel Index ≥ 5), age ≥ 65 years, and psychological distress at discharge (Hospital Anxiety and Depression Scale ≥ 8). **Results:** Global functional decline at three months was observed in 45% of patients. In logistic regression analysis, HAD (OR = 1.80, 95% CI: 1.00–3.24, *p* = 0.049), psychological distress (OR = 2.11, 95% CI: 1.27–3.49, *p* = 0.004), and older age (OR = 1.03 per year, *p* = 0.027) were independently associated with the outcome. Relative risk analysis confirmed similar associations: HAD (RR = 1.99, 95% CI: 1.71–2.31), psychological distress (RR = 1.35), and their combination significantly increased the risk of functional decline. Among patients who were ADL-independent at discharge (Barthel Index ≥ 85), those with all three risk factors had a markedly elevated risk (RR = 10.17, 95% CI: 6.46–16.00, *p* < 0.001). **Conclusions:** HAD, older age, and psychological distress at discharge are robust predictors of functional decline after ICU discharge, even in patients who appear functionally independent at discharge. These findings support comprehensive discharge planning that incorporates both physical and psychological assessments to identify high-risk individuals and improve long-term outcomes.

## 1. Introduction

Advancements in critical care have significantly improved survival rates among patients admitted to intensive care units (ICUs). However, many ICU survivors experience long-term impairments in physical, cognitive, and psychological functions, a condition widely recognized as post-intensive care syndrome (PICS) [1,2]. PICS negatively affects quality of life, independence in daily living, and return to societal roles, while increasing risks of rehospitalization and long-term care needs [3,4]. Consequently, the focus of critical care has shifted from short-term survival to long-term recovery and functional reintegration [5].

One of the major contributors to PICS is hospital-associated disability (HAD), which refers to a decline in activities of daily living (ADL) that develops during hospitalization in previously independent individuals [6]. HAD frequently results from immobility, enforced bed rest, and acute illness-related deconditioning. Elderly patients and those with preexisting chronic conditions are particularly vulnerable. In ICU settings, where the severity of illness is high and invasive procedures are standard, the risk of HAD is especially elevated [7,8].

Although some cases of HAD are transient, many patients do not fully recover their function after discharge. HAD is associated with greater dependency, fall risk, depression, and social isolation [6,9]. Early mobilization during the ICU stay has been shown to preserve physical function and reduce ICU-acquired weakness [10]. However, the incidence of HAD remains high in real-world clinical settings, and large-scale studies investigating its impact on post-discharge functional status are limited.

In this context, we aimed to investigate the relationship between in-hospital ADL decline (i.e., HAD) and functional impairment at 3 months after discharge using data from a nationwide, multicenter, prospective cohort study (J-RELIFE) involving 22 ICUs across Japan. Specifically, we focused on changes in the Barthel Index (BI) from ICU admission to discharge and their association with physical, cognitive, and psychological outcomes. We hypothesized that patients who experienced HAD during ICU stay would exhibit significantly higher rates of impaired functional status at three months post-discharge, even if they appeared functionally recovered at the time of hospital discharge. Establishing HAD as a prognostic marker, rather than a transient event, may support the development of targeted rehabilitation and discharge planning strategies.

## 2. Materials and Methods

### 2.1. Study Design and Setting

This study was a multicenter, prospective, observational cohort study conducted within the J-RELIFE project (UMIN000036503). The study was designed to investigate PICS, including physical, cognitive, and psychological impairments, and their associated risk factors in critically ill patients discharged from ICUs. Twenty-two tertiary hospitals with established ICU rehabilitation protocols participated in the study between October 2021 and December 2023. The study protocol was approved by the institutional review boards of all participating facilities, with central approval obtained from the Fujita Health University Ethics Committee (Approval ID: HM21-077). Written informed consent was obtained from all patients or their legally authorized representatives.

### 2.2. Participants

Eligible participants were adult patients (aged 40 years or older) admitted to the ICU who received mechanical ventilation for 48 h or more and were deemed eligible for ICU rehabilitation according to standard institutional criteria. The J-RELIFE study was designed to evaluate the implementation of rehabilitation in ICU settings. Therefore, only patients who received at least one rehabilitation session during their ICU stay were enrolled prospectively. Patients who did not undergo rehabilitation were excluded from the study cohort, in accordance with the predefined inclusion criteria. Patients were excluded if they (1) had central nervous system disorders causing disability; (2) had difficulty communicating in Japanese or had psychiatric conditions interfering with rehabilitation; (3) were unable to walk even with assistive devices before admission; (4) were under end-of-life care or died during hospitalization; or (5) were placed under COVID-19 quarantine, declined participation, had motor impairments that precluded walking assessment, or did not receive any rehabilitation sessions during their ICU stay.

### 2.3. Rehabilitation and Data Collection

All participating ICUs followed a standardized mobilization protocol categorized into five activity levels: level 1 (passive movement and respiratory therapy), level 2 (active range of motion), level 3 (sitting at bedside), level 4 (standing), and level 5 (ambulation) [11,12]. Rehabilitation was initiated as early as clinically feasible and provided daily when possible. Trained physiotherapists documented the start date of rehabilitation and the average daily duration.

Data were prospectively collected from ICU admission to three months post-discharge, including baseline demographics, comorbidities, ICU treatments, rehabilitation parameters, and functional assessments. Post-discharge follow-up was conducted using mailed questionnaires, including the Kihon Checklist (KCL), a validated screening tool for physical, cognitive, and psychological function in older adults [13].

### 2.4. Outcome Measures

The primary outcome of this study was functional status three months after hospital discharge, assessed using the KCL. A total KCL score ≥ 8 was defined as global functional decline. Additionally, the prevalence of impairment in the individual KCL domains—physical function, nutritional status, oral function, cognitive function, and depression—was assessed. These domains represent key components of long-term frailty and disability in ICU survivors. The age threshold of 40 years was selected because the primary outcome measure is the KCL, a validated screening tool developed for use in middle-aged and older adults in Japan [13]. Prior studies have demonstrated its predictive value for frailty, disability, and long-term care needs in this population. Moreover, functional decline following critical illness is not limited to the elderly but can also occur in adults from early middle age, especially among those with comorbidities. Therefore, including patients aged 40 years and older was deemed appropriate to capture both early- and late-onset post-ICU impairments while ensuring the applicability of the KCL.

Secondary outcomes included physical and psychological function at hospital discharge. Functional ability was assessed using the BI, the Short Physical Performance Battery (SPPB) [14], the Medical Research Council (MRC) score [15], and ambulatory status (independent, modified independent, dependent, or non-ambulatory). Psychological function was measured using the Hospital Anxiety and Depression Scale (HADS) [16] and the Impact of Event Scale-Revised (IES-R) [17]. HAD was defined as a decrease of ≥5 points in the BI from the pre-hospital baseline (assessed at ICU admission via patient or proxy interview) to hospital discharge [18,19]. Additional variables included ICU and hospital length of stay, mechanical ventilation duration, time to initiation of rehabilitation, and time to achievement of key mobilization milestones (sitting, standing, walking).

### 2.5. Covariates

We included clinically relevant covariates previously associated with functional outcomes in critically ill patients. Demographic variables included age and sex. To account for baseline disease burden and acute illness severity, we used the Charlson Comorbidity Index and APACHE II score at the time of ICU admission. Pre-hospital frailty was assessed using the Clinical Frailty Scale [20].

Two discharge-related variables were also included: (1) ADL dependency at discharge, defined as a BI score < 85 [19], and (2) psychological symptoms at discharge, defined by the presence of depression and/or anxiety according to the HADS [16]. These covariates represent biological, functional, and psychological domains that may affect post-discharge recovery.

### 2.6. Statistical Analysis

Analyses were performed on complete cases, including only patients with available 3-month follow-up Kihon Checklist (KCL) data. Missing data was not imputed. All statistical analyses were conducted using R software (version 4.0.5; R Foundation for Statistical Computing, Vienna, Austria). Descriptive statistics were used to summarize baseline characteristics. Continuous variables were reported as medians with interquartile ranges (IQRs) and compared using the Mann–Whitney U test. Categorical variables were presented as counts and percentages and compared using Fisher’s exact test. To identify independent predictors of functional decline at three months post-discharge, multivariable logistic regression analysis was performed. Covariates included age, sex, Charlson Comorbidity Index, APACHE II score, Clinical Frailty Scale, ADL dependency at discharge, and psychological symptoms at discharge. Adjusted odds ratios (AORs) with 95% confidence intervals (CIs) were reported. To examine associations with all-cause mortality three months after ICU discharge, we conducted a relative risk analysis using a generalized linear model with a log link and binomial distribution. The same covariates were included to estimate adjusted relative risks (RRs) and 95% confidence intervals.

## 3. Results

### 3.1. Baseline Characteristics

**Figure 1:**
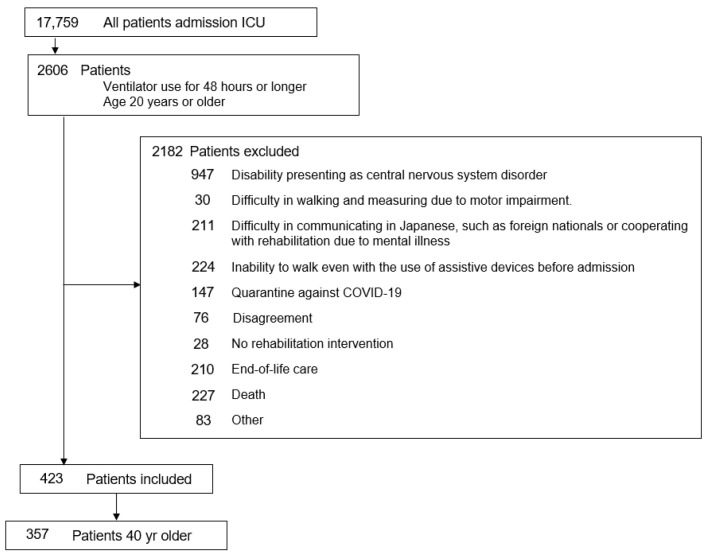
Flow chart of our study. ICU, Intensive Care Unit.

Of 17,759 ICU admissions, 2606 patients aged ≥ 20 years who received mechanical ventilation for ≥48 h were screened. After excluding 2182 patients due to clinical or functional limitations, 423 were eligible. Among them, 66 patients aged < 40 years were excluded, leaving 357 patients in the final analysis (Figure 1).

Table 1 shows the baseline characteristics of 357 ICU survivors, stratified by the presence (n = 260) or absence (n = 97) of functional decline at 3 months post-discharge. Patients in the decline group were significantly older than those in the non-decline group (*p* < 0.001) and had higher Charlson Comorbidity Index scores (*p* < 0.001). The Clinical Frailty Scale was also higher in the decline group (*p* = 0.014), despite comparable pre-admission BI scores.

Rehabilitation milestones were delayed in the decline group, as evidenced by significantly longer times to first sitting (*p* = 0.008), standing (*p* = 0.002), and marching (*p* = 0.004) compared to the non-decline group. The rehabilitation time per session was also slightly shorter in the decline group (*p* = 0.036). Time to initiate rehabilitation was modestly delayed in the decline group (*p* = 0.045) (Table 1).

### 3.2. Primary Outcomes

Table 2 presents the prevalence of impairments across six domains of the Kihon Checklist assessed at 3 months after hospital discharge. At this point, global functional decline—the primary outcome of the study —was defined as a total KCL score of 8 or greater and was observed in 72.8% of patients. The most frequent individual impairment was cognitive function decline (64.4%), followed by possible depression (48.2%) and physical function decline (47.3%). Declines in oral function and nutritional status were also noted in 28.3% and 15.7% of patients, respectively (Table 2).

### 3.3. Secondary Outcomes

Table 3 summarizes the physical and psychological status of patients at hospital discharge, stratified by functional outcome at 3 months. Patients in the functional decline group had significantly lower BI scores (median 95 vs. 100, *p* < 0.001), lower MRC scores (58 vs. 60, *p* = 0.015), and lower SPPB scores (10 vs. 11, *p* = 0.004), particularly in the chair stand (2 vs. 4, *p* = 0.001) and gait speed tests (4 vs. 4, *p* = 0.037). Psychological assessments also revealed significantly higher HADS in the decline group, with 50.8% scoring ≥ 8 for depression and 40.0% for anxiety, compared to 29.9% and 24.7%, respectively, in the non-decline group (*p* < 0.01). PTSD symptoms (IES-R ≥ 25) were more frequent in the decline group (21.9% vs. 11.3%, *p* = 0.023). Additionally, functional decline during hospitalization (ΔBI ≥ 5) was more prevalent in the decline group (42.0% vs. 21.6%, *p* < 0.001), and fewer were fully ambulatory at discharge (65.4% vs. 79.4%, *p* = 0.014).

### 3.4. Multivariable Analysis

Table 4 shows the results of multivariable logistic regression analyses examining factors associated with functional decline at 3 months post-discharge. In Model 1, discharge ADL dependency (BI < 85) was significantly associated with functional decline (OR = 2.45, 95% CI: 1.24–4.82, *p* = 0.009), as well as older age (*p* = 0.004). In Model 2, HAD was a significant predictor (OR = 2.17, 95% CI: 1.23–3.83, *p* = 0.007), and the Charlson Comorbidity Index also reached statistical significance (OR = 1.18, 95% CI: 1.01–1.38, *p* = 0.042). In both models, depression or anxiety at discharge remained significantly associated with functional decline (ORs ~2.1, *p* < 0.01). In Model 3, which included all variables, HAD (OR = 1.80, 95% CI: 1.00–3.24, *p* = 0.049) and depression/anxiety (OR = 2.11, 95% CI: 1.27–3.49, *p* = 0.004) remained independently associated with the outcome, whereas the effect of discharge ADL dependency was no longer statistically significant (OR = 1.88, *p* = 0.077). Age was a consistent predictor across all models (*p* < 0.05) (Table 4).

Table 5 presents the results of the relative risk analysis for global functional decline at 3 months after hospital discharge. In the overall cohort (n = 357), hospital-acquired disability (HAD) was significantly associated with increased risk of decline (RR = 1.99, 95% CI: 1.71–2.31, *p* < 0.001). Older age (≥65 years) and psychological symptoms at discharge (depression and/or anxiety) were also independent risk factors (RR = 1.13 and 1.35, respectively; both *p* < 0.05). Patients with both HAD and psychological symptoms had a combined risk of 3.02 (95% CI: 2.49–3.67), while those with all three factors—HAD, older age, and psychological symptoms—had the highest observed risk (RR = 3.66, 95% CI: 2.95–4.55, *p* < 0.001). Additionally, ADL dependency at discharge (Barthel Index < 85) was strongly associated with functional decline (RR = 2.92, 95% CI: 2.41–3.54, *p* < 0.001). In the subgroup of patients who were functionally independent at discharge (BI ≥ 85, n = 268), similar associations were observed. HAD remained a robust predictor (RR = 3.98, 95% CI: 3.02–5.24, *p* < 0.001), and the presence of all three risk factors conferred a markedly elevated risk of decline (RR = 10.17, 95% CI: 6.46–16.00, *p* < 0.001) (Table 5).

## 4. Discussion

In this multicenter prospective cohort study, we found that HAD, older age, and psychological distress at discharge were independently associated with global functional decline three months after discharge in ICU survivors. Notably, HAD alone was associated with a 4-fold increase in the odds of functional decline, and the cumulative presence of all three risk factors was associated with a 10-fold increase. These findings underscore the importance of monitoring and addressing physical and psychological vulnerabilities in critically ill patients not only during ICU stay, but also throughout the transition to post-acute care.

Our findings are consistent with previous reports that have highlighted HAD as a key consequence of critical illness, especially in older adults and those undergoing prolonged immobilization [6,7]. HAD, characterized by a decline in ADL during hospitalization despite previously independent status, has been associated with increased mortality, readmission, and long-term care needs [19]. While early mobilization during the ICU stay has been shown to mitigate the risk of HAD [10,11], our study suggests that even when patients regain basic ADL independence at discharge, a history of in-hospital functional decline may continue to influence post-discharge outcomes. This highlights the limitation of relying solely on discharge BI or SPPB scores when estimating long-term prognosis. These results aligned with previous reports highlighting the long-term impact of HAD in ICU survivors. For example, Schweickert et al. [10] demonstrated that early mobilization during ICU stay improved short-term physical outcomes but did not fully prevent long-term functional limitations. Herridge et al. [21] reported persistent physical disability lasting up to five years after ICU discharge, even in younger patients. These earlier studies underscored the prolonged recovery trajectory following critical illness but focused primarily on physical performance over time. Compared to those studies, our cohort was restricted to adults aged ≥ 40 years who were independent before ICU admission and received structured rehabilitation, allowing us to assess HAD as a discharge-time phenomenon more precisely. Additionally, while Schweickert et al. emphasized early interventions [10] and Herridge et al. tracked long-term outcomes [21], our study uniquely identifies discharge-time markers—such as HAD and psychological distress—that predict medium-term (3-month) global functional decline. Our use of the BI as a standardized ADL measure also allows for direct quantification of hospital-acquired changes, which some prior studies did not assess in detail.

Furthermore, the synergistic effect observed between HAD, older age, and depression/anxiety suggests a biopsychosocial interaction that shapes recovery trajectories after critical illness. The co-occurrence of HAD, psychological distress, and older age suggests a complex interplay between physiological vulnerability, emotional resilience, and aging-related decline. The presence of psychological symptoms at discharge may compound physical vulnerability, leading to disengagement from rehabilitation or reduced self-efficacy in resuming daily activities. Similarly, frail older adults may lack the physiological reserve to recover from acute functional losses [20]. These findings align with the PICS framework, which describes persistent impairments across physical, cognitive, and mental domains after ICU discharge [4,22].

From a clinical standpoint, our findings support the need for more nuanced discharge planning that incorporates both objective physical performance measures and psychological status assessments. Patients with HAD, even if functionally independent at discharge, should be prioritized for early and structured post-discharge rehabilitation and follow-up. Integrating frailty screening and mood assessment into discharge protocols may improve the targeting of such interventions. Standard discharge checklists could be enhanced by incorporating screening for HAD and psychological symptoms. Moreover, patients with multiple risk factors may benefit from integrated care pathways with a long-term perspective, coordinated by interdisciplinary teams composed of physiotherapists, occupational therapists, mental health professionals, social workers, physicians, and nurses.

## 5. Limitations

Several limitations of our study warrant consideration. First, although the multicenter design enhances the external validity of our findings, differences in rehabilitation protocols across institutions may have introduced variability. While all participating centers adhered to standardized rehabilitation procedures, minor institutional variations likely remained and could have influenced patient outcomes. This potential confounding should be acknowledged when interpreting the results. Second, functional outcomes were assessed using self-reported questionnaires, which may be subject to response bias. These tools are also self-reported, raising concerns about reporting bias and potential under- or over-estimation of psychological distress. Similarly, pre-hospital ADL was assessed retrospectively via interview, which may be subject to recall bias, potentially limiting the accuracy of baseline functional status. Third, unmeasured factors such as nutritional status, social support, and psychosocial interventions may have influenced recovery. Future studies incorporating these aspects will be essential to provide a more comprehensive understanding of post-ICU recovery. As this was an observational study, our findings describe associations rather than causal relationships. Furthermore, although relative risk analysis was used to examine associations with functional decline, the outcome was assessed at a single time point (three months post-discharge), and the exact timing of the decrease was not recorded. As such, the estimated risk ratios should be interpreted as approximations of relative risk rather than actual time-to-event effects. While the Kihon Checklist is validated for middle-aged and older adults, excluding ICU survivors under 40 years of age may introduce selection bias and limit the generalizability of our findings to younger populations. Younger adults may have different patterns of recovery and risk factors for functional decline. Future studies should consider including validated outcome measures suitable for younger ICU survivors to assess post-ICU trajectories across age groups more comprehensively. Finally, our exclusion of patients with pre-existing central nervous system disorders, psychiatric illness, or communication difficulties, while necessary for accurate HAD classification, may have introduced selection bias. These populations are known to be at increased risk of post-ICU impairment, and their exclusion may lead to underestimation of the true prevalence and burden of functional decline after critical illness. Despite these limitations, this study adds necessary evidence to the growing body of literature on post-ICU outcomes. It supports conceptualizing HAD as a dynamic risk factor—not merely a transient in-hospital event—but as a long-term predictor of global functional decline. These insights may guide more effective transitional care models and rehabilitation strategies aimed at reducing disability and promoting recovery among ICU survivors.

## 6. Conclusions

This multicenter prospective cohort study demonstrated that HAD, older age, and psychological distress at discharge are independent and cumulative predictors of global functional decline at three months after hospital discharge in ICU survivors. Notably, even patients who recovered their ADL independence by discharge remained at increased risk of post-discharge decline if they had experienced HAD during hospitalization. These findings emphasize the importance of not only early mobilization in the ICU but also comprehensive discharge assessment that includes physical, psychological, and frailty-related factors. Identifying high-risk patients at discharge may facilitate timely interventions and structured rehabilitation programs to mitigate long-term disability and enhance post-ICU recovery trajectories. Future research should focus on intervention strategies tailored to individuals with HAD and explore the effectiveness of multidisciplinary post-discharge care pathways.

## Figures and Tables

**Table 1:** Baseline characteristics of ICU survivors stratified by the presence or absence of functional decline at 3 months post-discharge.

	All Patients (N = 357)		Decline (n = 260)		Decline (n = 97)		*p*-Value
	Median, IQR, or n (%)		Median, IQR, or n (%)		Median, IQR, or n (%)		
Age, yr	71	59–76	72.5	61.75–78	64	54–74	<0.001
Male, n (%)	233	65.2%	167	64.20%	66	68.00%	0.534
Body mass index, kg/m^2^	24	21.4–27.5	24	21.2–27.4	24.6	22.0–27.8	0.250
Charlson comorbidity index	1	0–2	1	0–2	0	0–2	<0.001
Emergency case, n (%)	279	78.2%	205	78.80%	74	76.30%	0.666
SOFA total score, score	10	6–13	10	6–12	10	7–13	0.304
APACHE II (on admission)	21	14–27	20	14.5–26.0	22	13.0–28.25	0.202
Clinical Frailty Scale, score	3	2–3	3	2–3	2	2–3	0.014
Barthel index, pre-admission, score	100	100–100	100	100–100	100	100–100	0.484
Length of ICU stay, day	7.2	5.1–11.3	7.53	5.33–11.5	6.595	4.84–9.72	0.131
Mechanical ventilator duration, day	4.6	3.0–8.4	4.65	3.04–8.00	3.89	2.94–7.28	0.199
Rehabilitation, period to start, day	1.0	0.7–2.0	1.03	0.77–2.02	0.89	0.61–1.71	0.045
Sitting, period to start, day	3.7	2.1–5.9	3.8	2.2–6.3	3.0	1.8–4.8	0.008
Standing, period to start, day	5.2	3.6–8.7	5.7	3.7–9.4	4.1	2.7–6.7	0.002
Marching, period to start, day	6.9	4.5–10.9	7.4	4.8–11.5	5.2	3.7–9.2	0.004
Rehabilitation min/time, min	32.3	20–44	31.6	20.00%	35	25.0–46.7	0.036

Data are presented as median (IQR) or number (%). *p*-values were calculated using the Mann–Whitney U test or Fisher’s exact test, as appropriate. Abbreviations: SOFA, Sequential Organ Failure Assessment; APACHE II, Acute Physiology and Chronic Health Evaluation II; ICU, intensive care unit; IQR, Interquartile Range.

**Table 2:** Prevalence of individual components of the Kihon Checklist at 3 months after hospital discharge.

Kihon Checklist Domains	Impairment Prevalence, n (%)
Physical function decline	169 (47.3%)
Nutrition risk	56 (15.7%)
Oral function decline	101 (28.3%)
Cognitive function decline	230 (64.4%)
Possible depression	172 (48.2%)
Global functional decline (KCL ≥ 8)	260 (72.8%)

Data are presented as numbers (%). Impairments were defined according to standard Kihon Checklist scoring criteria. Global functional decline was defined as a total KCL score ≥ 8.

**Table 3:** Physical and psychological function at hospital discharge in ICU survivors with and without functional decline at 3 months.

Hospital Discharge	Decline (n = 260)		Non-Decline (n = 97)		*p*-Value
	Median, IQR, or n (%)		Median, IQR, or n (%)		
Length of hospital stay, day	36.1	24.4–53.6	32.5	25.1–60.9	0.699
Barthel index, score	95	75–100	100	90–100	<0.001
MRC score	58	51–60	60	55–60	0.015
SPPB, score	10	6–12	11	8–12	0.004
Balance test, score	4	3–4	4	3–4	0.123
Gait speed test, score	4	2–4	4	3–4	0.037
Chair stand test, score	2	0–4	4	2–4	0.001
SPPB total score ≤ 9, n (%)	112	43.1%	28	28.9%	0.015
HADs, depression, score	8	4–11	5	2–9	<0.001
≥8, n (%)	132	50.8%	29	29.9%	<0.001
HADs, anxiety, score	7	4–10	4	1–8	<0.001
≥8, n (%)	104	40.00%	24	24.70%	0.009
IES-R, score	13	6–24	6	2–13	<0.001
≥25, n (%)	57	21.9%	11	11.3%	0.023
Functional decline (ΔBI ≥ 5)	110	42.0%	21	21.6%	<0.001
Ambulation					
Independent, n (%)	170	65.4%	77	79.4%	0.014
Modified independence, n (%)	38	14.6%	13	13.4%	0.866
Dependent, n (%)	37	14.2%	4	4.1%	0.008
Non-ambulation, n (%)	15	5.8%	3	3.1%	0.419

Data are presented as median (IQR) or number (%). *p*-values were calculated using the Mann–Whitney U test or Fisher’s exact test, as appropriate. Abbreviations: BI, Barthel Index; HADS, Hospital Anxiety and Depression Scale; IES-R, Impact of Event Scale-Revised; MRC, Medical Research Council scale; SPPB, Short Physical Performance Battery; IQR, Interquartile Range.

**Table 4:** Multivariable logistic regression analysis of risk factors for functional decline at 3 months post-discharge.

Model 1				Model 2				Model 3			
	OR	95%CI	*p*-value		OR	95%CI	*p*-value		OR	95%CI	*p*-value
Age	1.03	1.01–1.04	0.004	Age	1.02	1.01–1.04	0.009	Age	1.03	1.01–1.05	<0.001
Male	0.982	0.58–1.64	0.944	Male	0.92	0.54–1.56	0.764	Male	0.914	0.54–1.56	0.742
Dependent (BI < 85)	2.45	1.24–4.82	0.009	Dependent (BI < 85)	2.22	1.12–4.41	0.023	Dependent (BI < 85)	1.88	0.94–3.8	0.077
				Charlson comorbidity index	1.16	0.99–1.36	0.061	Charlson comorbidity index	1.15	0.99–1.35	0.072
				APACHE II	0.98	0.94–1.01	0.11	APACHE II	0.975	0.95–1.00	0.096
				Clinical Frailty Scale	1.09	0.85–1.38	0.503	Clinical Frailty Scale	1.07	0.84–1.36	0.591
								Depression & Anxiety	2.12	1.28–3.51	0.003
	OR	95%CI	*p*-value		OR	95%CI	*p*-value		OR	95%CI	*p*-value
Age	1.02	1.01–1.04	0.007	Age	1.02	1.00–1.04	0.017	Age	1.02	1.01–1.04	0.012
Male	1.03	0.61–1.74	0.902	Male	0.97	0.57–1.65	0.91	Male	0.95	0.55–1.63	0.853
HAD (ΔBI ≥ 5)	2.17	1.23–3.83	0.007	HAD (ΔBI ≥ 5)	2.06	1.16–3.67	0.013	HAD (ΔBI ≥ 5)	1.8	1.00–3.24	0.049
				Charlson comorbidity index	1.18	1.01–1.38	0.042	Charlson comorbidity index	1.17	0.997–1.36	0.055
				APACHE II	0.98	0.94–1.01	0.115	APACHE II	0.98	0.95–1.01	0.105
				Clinical Frailty Scale	1.09	0.85–1.38	0.483	Clinical Frailty Scale	1.07	0.84–1.37	0.565
								Depression & Anxiety	2.11	1.27–3.49	0.004

Odds ratios (ORs), 95% confidence intervals (CIs), and *p*-values from multivariable logistic regression models evaluating associations with functional decline (KCL > 8) at 3 months after discharge. Model 1 includes ADL dependency at discharge (BI < 85), Model 2 includes hospital-acquired disability, and Model 3 includes all variables. Abbreviations: APACHE II, Acute Physiology and Chronic Health Evaluation II; BI, Barthel Index; HAD, hospital-acquired disability; KCL, Kihon Checklist.

**Table 5:** Risk Ratios for 3-Month Mortality After ICU Discharge by Clinical Indicators.

All Patients, n = 357	RR	95% CI	*p*-Value
Older patients (≥65)	1.126	1.019–1.243	0.024
Depression/Anxiety	1.354	1.207–1.519	<0.001
Older patients + Depression/Anxiety	2.031	1.744–2.366	<0.001
HAD	1.985	1.708–2.307	<0.001
HAD + Older patients	2.407	2.032–2.853	<0.001
HAD + Depression/Anxiety	3.023	2.488–3.673	<0.001
HAD + Older patients + Depression/Anxiety	3.662	2.946–4.552	<0.001
Dependent (BI < 85)	2.921	2.414–3.536	<0.001
Independent (BI ≥ 85), n = 268	RR	95% CI	*p*-value
Older patients (≥65)	1.173	1.03–1.336	0.02
Depression/Anxiety	1.419	1.223–1.646	<0.001
Older patients + Depression/Anxiety	2.473	2.004–3.052	<0.001
HAD	3.978	3.021–5.239	<0.001
HAD + Older patients	5.083	3.711–6.963	<0.001
HAD + Depression/Anxiety	7.625	5.161–11.266	<0.001
HAD + Older patients + Depression/Anxiety	10.167	6.459–16.002	<0.001

Relative risks (RRs), 95% confidence intervals (CIs), and *p*-values are shown for combinations of hospital-acquired disability (HAD), age ≥ 65, and depression/anxiety at discharge. Abbreviations: ADL, activities of daily living; BI, Barthel Index; CI, confidence interval; HAD, hospital-acquired disability.

## Data Availability

Data available on request due to restrictions (e.g., privacy, legal or ethical reasons). The data presented in this study are available upon request from the corresponding author due to ethical restrictions on patient confidentiality.

## Abbreviations

The following abbreviations are used in this manuscript:

ADL	Activities of Daily Living
ICU	Intensive Care Unit
PICS	Post-Intensive Care Syndrome
BI	Bathel Index
J-ReLIFE	Japanese Research on Rehabilitation and Risk Factors in Post-Intensive Care Patients
KCL	Kihon Checklist
HAD	Hospital-Acquired Disability
SPPB	Short Physical Performance Battery
MRC	Medical Research Council
HADS	Hospital Anxiety and Depression Scale
IES-R	Impact of Event Scale-Revised
IQR	Interquartile Range
APACHE II	Acute Physiology and Chronic Health Evaluation II
HR	Hazard Ratio
AOR	Adjusted Odds Ratio
CI	Confidence Interval

## References

[1] Needham D.M., Davidson J., Cohen H., Hopkins R.O., Weinert C., Wunsch H., Christine Z., Anita B.-D., Berney S.C., Joseph B.O. (2012). Improving long-term outcomes after discharge from intensive care unit: Report from a stakeholders’ conference. Crit. Care Med..

[2] Rawal G., Yadav S., Kumar R. (2017). Post-intensive care syndrome: An overview. J. Transl. Int. Med..

[3] Pandharipande P.P., Girard T.D., Jackson J.C., Morandi A., Thompson J.L., Pun B.T., Brummel N.E., Hughes C.G., Vasilevskis E.E., Shintani A.K. (2013). Long-term cognitive impairment after critical illness. N. Engl. J. Med..

[4] Marra A., Pandharipande P.P., Girard T.D., Patel M.B., Hughes C.G., Jackson J.C., Thompson J.L., Chandrasekhar R., Ely E.W., Brummel N.E. (2018). Co-occurrence of post-intensive care syndrome problems among 406 survivors of critical illness. Crit. Care Med..

[5] Iwashyna T.J. (2010). Survivorship will be the defining challenge of critical care in the 21st century. Ann. Intern. Med..

[6] Covinsky K.E., Pierluissi E., Johnston C.B. (2011). Hospitalization-associated disability: “She was probably able to ambulate, but I’m not sure”. JAMA.

[7] Zisberg A., Shadmi E., Gur-Yaish N., Tonkikh O., Sinoff G. (2015). Hospital-associated functional decline: The role of hospitalization processes beyond individual risk factors. J. Am. Geriatr. Soc..

[8] Hermans G., Van Mechelen H., Clerckx B., Vanhullebusch T., Mesotten D., Wilmer A., Casaer M.P., Meersseman P., Debaveye Y., Van Cromphaut S. (2014). Acute outcomes and 1-year mortality of intensive care unit-acquired weakness. A cohort study and propensity-matched analysis. Am. J. Respir. Crit. Care Med..

[9] Fan E., Dowdy D.W., Colantuoni E., Mendez-Tellez P.A., Sevransky J.E., Shanholtz C., Himmelfarb C.R.D., Desai S.V., Ciesla N., Herridge M.S. (2014). Physical complications in acute lung injury survivors: A two-year longitudinal prospective study. Crit. Care Med..

[10] Schweickert W.D., Pohlman M.C., Pohlman A.S., Nigos C., Pawlik A.J., Esbrook C.L., Spears L., Miller M., Franczyk M., Deprizio D. (2009). Early physical and occupational therapy in mechanically ventilated, critically ill patients: A randomised controlled trial. Lancet..

[11] Morris P.E., Goad A., Thompson C., Taylor K., Harry B., Passmore L., Ross A., Anderson L., Baker S., Sanchez M. (2008). Early intensive care unit mobility therapy in the treatment of acute respiratory failure. Crit. Care Med..

[12] Watanabe S., Liu K., Nakamura K., Kozu R., Horibe T., Ishii K., Yasumura D., Takahashi Y., Nanba T., Morita Y. (2022). Association between early mobilization in the ICU and psychiatric symptoms after surviving a critical illness: A multi-center prospective cohort study. J. Clin. Med..

[13] Watanabe D., Yoshida T., Watanabe Y., Yamada Y., Miyachi M., Kimura M. (2022). Validation of the Kihon Checklist and the frailty screening index for frailty defined by the phenotype model in older Japanese adults. BMC Geriatr..

[14] Guralnik J.M., Simonsick E.M., Ferrucci L., Glynn R.J., Berkman L.F., Blazer D.G., Scherr P.A., Wallace R.B. (1994). A short physical performance battery assessing lower extremity function: Association with self-reported disability and prediction of mortality and nursing home admission. J. Gerontol..

[15] Kleyweg R.P., van der Meché F.G., Schmitz P.I. (1991). Interobserver agreement in the assessment of muscle strength and functional abilities in Guillain–Barré syndrome. Muscle Nerve..

[16] Zigmond A.S., Snaith R.P. (1983). The hospital anxiety and depression scale. Acta Psychiatr. Scand..

[17] Beck J.G., Grant D.M., Read J.P., Clapp J.D., Coffey S.F., Miller L.M., Palyo S.A. (2008). The Impact of Event Scale-Revised: Psychometric properties in a sample of motor vehicle accident survivors. J. Anxiety Disord..

[18] Takahashi Y., Morisawa T., Okamoto H., Matsumoto N., Saitoh M., Takahashi T., Fujiwara T. (2023). Relationship Between Skeletal Muscle Quality and Hospital-Acquired Disability in Patients with Sepsis Admitted to the ICU: A Pilot Study. Crit. Care Explor..

[19] Church S., Rogers E., Rockwood K., Theou O. (2020). A scoping review of the Clinical Frailty Scale. BMC Geriatr..

[20] Katano S., Yano T., Ohori K., Kouzu H., Nagaoka R., Honma S., Shimomura K., Inoue T., Takamura Y., Ishigo T. (2021). Barthel Index score predicts mortality in elderly heart failure—A goal of comprehensive cardiac rehabilitation. Circ. J..

[21] Herridge M.S., Tansey C.M., Matté A., Tomlinson G., Diaz-Granados N., Cooper A., Guest C.B., Mazer C.D., Mehta S., Stewart T.E. (2011). Functional disability 5 years after acute respiratory distress syndrome. N. Engl. J. Med..

[22] Inoue S., Hatakeyama J., Kondo Y., Hifumi T., Sakuramoto H., Kawasaki T., Taito S., Nakamura K., Unoki T., Kawai Y. (2019). Post-intensive care syndrome: Its pathophysiology, prevention, and future directions. Acute Med. Surg..

